# High-Density Lipoprotein-Targeted Therapies for Heart Failure

**DOI:** 10.3390/biomedicines8120620

**Published:** 2020-12-16

**Authors:** Mudit Mishra, Bart De Geest

**Affiliations:** Center for Molecular and Vascular Biology, Department of Cardiovascular Sciences, Catholic University of Leuven, 3000 Leuven, Belgium; drmuditm@gmail.com

**Keywords:** high-density lipoproteins, heart failure, cardiac hypertrophy, myocardial fibrosis, gene therapy, nanoparticles, heart failure with preserved ejection fraction, apolipoprotein A-I

## Abstract

The main and common constituents of high-density lipoproteins (HDLs) are apolipoprotein A-I, cholesterol, and phospholipids. Biochemical heterogeneity of HDL particles is based on the variable presence of one or more representatives of at least 180 proteins, 200 lipid species, and 20 micro RNAs. HDLs are circulating multimolecular platforms that perform divergent functions whereby the potential of HDL-targeted interventions for treatment of heart failure can be postulated based on its pleiotropic effects. Several murine studies have shown that HDLs exert effects on the myocardium, which are completely independent of any impact on coronary arteries. Overall, HDL-targeted therapies exert a direct positive lusitropic effect on the myocardium, inhibit the development of cardiac hypertrophy, suppress interstitial and perivascular myocardial fibrosis, increase capillary density in the myocardium, and prevent the occurrence of heart failure. In four distinct murine models, HDL-targeted interventions were shown to be a successful treatment for both pre-existing heart failure with reduced ejection fraction (HFrEF) and pre-existing heart failure with preserved ejection fraction (HFrEF). Until now, the effect of HDL-targeted interventions has not been evaluated in randomized clinical trials in heart failure patients. As HFpEF represents an important unmet therapeutic need, this is likely the preferred therapeutic domain for clinical translation.

## 1. Introduction: Heart Failure, High-Density Lipoproteins (HDLs), and HDL-Targeted Therapies

Heart failure is a clinical syndrome distinguished by characteristic symptoms (e.g., shortness of breath, ankle swelling, and fatigue) that may be accompanied by clinical signs (e.g., elevated jugular venous pressure, pulmonary crackles, and peripheral edema) [1]. This syndrome is caused by a structural and/or functional cardiac abnormality, which results in a decreased cardiac output and/or increased intracardiac pressures at rest or during stress [1]. Systolic or diastolic dysfunction is not considered to constitute heart failure in asymptomatic subjects. The underlying cardiac cause of heart failure is most often a myocardial abnormality causing systolic and/or diastolic ventricular dysfunction, but in a minority of patients, heart failure arises secondary to pericardial pathology, endocardial pathology, valvular heart disease, heart rhythm disturbances, or conduction abnormalities [1]. The high-density lipoprotein (HDL)-targeted interventions for heart failure discussed in this review are specifically considered for heart failure secondary to myocardial pathology.

HDLs are defined as lipoproteins with a density between 1.063 and 1.21 g/mL. However, ultracentrifugation is a physically invasive method for the isolation of HDLs and several alternative methods are used. In addition, an important part of HDLs in the physiological sense of the word has a density higher than 1.21 g/mL like pre-β HDL particles [2]. The common and principal components of HDL particles are apolipoprotein (apo) A-I, cholesterol, and phospholipids. Biochemical heterogeneity of HDL particles is based on the variable presence of one or more representatives of at least 180 proteins, 200 lipid species, and 20 micro RNAs. However, the exact number of proteins in HDLs is far from certain. A recent comprehensive analysis of the literature concluded that more than 550 proteins have been reported to be present in HDLs, but only a small subset of these are consistently retrieved across multiple studies, even when an identical isolation technique was applied [3]. Functionally, HDLs are circulating multimolecular platforms that perform divergent functions such as reverse (centripetal) cholesterol transport to the liver, anti-inflammation, antioxidation, immunomodulation, protection of the endothelium, and enhancement of endothelial function. The functional properties of HDLs depend on the global proteome and lipidome of the particles and do not necessarily correlate with the HDL cholesterol concentration. In addition, the composition of HDLs can change according to clinical circumstances so that these properties are lost and that HDLs even exerts unwanted and harmful effects [4].

In this review, we will approach the topic of HDL-targeted therapies for heart failure from three different angles: biological mechanisms that may underlie the beneficial effects of HDL-targeted therapies in heart failure (Section 2), epidemiological studies on the relationship between HDLs and heart failure incidence and heart failure prognosis (Section 3), and animal intervention experiments demonstrating the efficacy of HDL-targeted therapies in several distinct models of non-ischemic heart failure (Section 4). The focus on non-ischemic heart failure in Section 4 does not imply that HDL-targeted therapies are not considered at all for heart failure secondary to ischemic cardiomyopathy [5]. In contrast, the protection by HDLs against ischemia/reperfusion injury resulting in a decrease of infarct size and thus in myocardial salvage [6,7] is only tangentially related to the topic of HDLs and heart failure and is therefore not discussed in this review. Similarly, the impact of HDLs on infarct expansion and on ventricular remodeling after myocardial infarction [8,9,10] is outside the scope of the current review. For a comprehensive review of these specific themes, we refer to Van Linthout et al. [10].

## 2. Biological Mechanisms That May Contribute to the Beneficial Effects of HDL-Targeted Therapies in Heart Failure

Scientific reductionism and biological plausibility per se constitute a weak and insufficient foundation for clinical translation. Therefore, the mechanisms of action presented in this section should be considered in light of the in vivo evidence of animal intervention studies in Section 4. 

HDL-targeted interventions might have direct effects on the myocardium [11,12]. Transendothelial transport of HDLs to the interstitium occurs via transcytosis [13]. These direct effects should be considered in light of existing knowledge on the cellular composition of the myocardium. Cardiomyocytes occupy roughly 70% to 85% of the volume of the mammalian heart [14,15] and constitute approximately 30% of the cells in the mammalian heart, whereas non-myocyte cells comprise 70% of cardiac cells [16,17]. Recent work from Pinto et al. [18] using several independent methodological approaches in mice has demonstrated that endothelial cells constitute 64%, hematopoietic-derived cells 9%, fibroblast 15%, and non-fibroblast resident mesenchymal cells 12% of nonmyocyte cells in the myocardium of mice. The non-fibroblast resident mesenchymal cells comprise vascular smooth muscle cells and pericytes. Based on immunohistochemical analysis of human cardiac tissue, Pinto et al. [18] demonstrated that 31% of nuclei in the human heart correspond to cardiomyocytes (α-actinin 2 positive cells), 54% to endothelial cells (CD31 positive cells), 3% to leukocytes (CD45 positive cells), and the remaining to resident mesenchymal cells including fibroblasts.

After discussing the direct effects of HDLs on cardiomyocytes, myocardial endothelial cells, and fibrosis, we will examine the potential role of the anti-inflammatory and antioxidative properties of HDLs. The impact of these properties of HDLs on the heart may be secondary to the local presence of HDLs in the myocardium or may reflect systemic effects of HDLs in non-cardiac tissues or on circulating cells.

### 2.1. Direct Effects of HDLs on Cardiomyocytes

Durham et al. [19] demonstrated that HDLs protect cardiomyocytes against necrosis induced by oxygen and glucose deprivation via a mechanism involving scavenger receptor class B, type 1 (SR-BI). HDL treatment resulted in the phosphorylation of protein kinase B (Akt) in cardiomyocytes in SR-BI^+/+^ cardiomyocytes, but not in SR-BI^−/−^ cardiomyocytes. Chemical inhibition of phosphatidylinositol-3-kinase (PI3K) or of Akt at the protein level and silencing of either Akt1 or Akt2 gene expression abrogated HDL-mediated protection against oxygen and glucose deprivation-induced necrosis of cardiomyocytes. Since myocardial capillary rarefaction is a feature of pathological hypertrophy [20] and since oxygen extraction in the myocardium is already very high at rest (60–80%), even in normal subjects [21], this mechanism of cardiomyocyte protection may therefore be operational in the setting of heart failure.

The angiotensin II type 1 (AT1) receptor plays a major role in the development of cardiac hypertrophy. Mechanical stress upregulated AT1 receptor expression in cardiomyocytes in vitro, whereas the addition of HDLs significantly suppressed AT1 receptor upregulation [22]. This biochemical effect was accompanied by inhibition of cardiomyocyte hypertrophy in vitro [22].

Frias et al. [23] reported that HDLs activate signal transducer and activator of transcription 3 (STAT3) in ventricular cardiomyocytes in vitro via extracellular signal-regulated kinases 1/2 and that sphingosine-1-phosphate, a constituent of HDLs, plays a major role in this activation. The Janus kinase/STAT pathway induces cardiomyocyte hypertrophy [24,25]. In contrast, the integrated effect of HDLs on cardiomyocytes in vitro is an inhibition of cardiomyocyte hypertrophy [22]. As will be discussed later, HDLs also inhibit cardiomyocyte hypertrophy in vivo. On the other hand, STAT3 activation by HDLs may have beneficial effects since STAT3 protects cardiomyocytes against apoptosis and since it plays a crucial role in cardiomyocyte resistance to inflammation [26].

Van Linthout et al. [27] demonstrated that HDLs have direct effects on contractility of cardiomyocytes obtained from Sprague Dawley rats. Under conditions of hyperglycemia, HDLs abrogated the glucose-induced reduction in contractility. This effect of HDLs was abolished in the presence of wortmannin, a PI3K inhibitor, or of the nitric oxide synthase inhibitor N^G^-nitro-L-arginine methyl ester (L-NAME). This is the most direct evidence using isolated cardiomyocytes that HDLs may result in significant physiological effects.

Reconstituted HDLs containing wild-type apo A-I has been demonstrated to shorten repolarization in cardiomyocytes isolated from rabbits [28]. Correspondingly, infusion of reconstituted HDLs in humans decreased the heart-rate corrected QT interval on surface electrocardiograms [28]. HDLs are an important regulator of the distribution of cholesterol between raft and non-raft membrane fractions [29]. Since microdomain-specific localization of ion channels affect their function [30], the impact of HDLs on the plasma membrane structure may underlie its impact on cardiomyocyte repolarization.

### 2.2. Direct Effects of HDLs on Myocardial Endothelial Cells

Endothelial cells line the vascular myocardial compartment. Since decreased coronary sinus oxygen content is a predictor of adverse prognosis in patients with severe congestive heart failure [31], the vascular compartment is a critical player in heart failure. As already highlighted *supra* [16,17,18], endothelial cells are the predominant cell type in the myocardium. The coronary circulation comprises a proximal compartment (the epicardial arteries or coronary macro-circulation with vessels ranging from 5.0 mm in diameter to 0.5 mm in diameter), an intermediate compartment with pre-arteriolar vessels (diameter between 0.5 mm and 0.1 mm), and a distal compartment with arterioles, which have a diameter less than 100 µm [32,33]. Together, the pre-arterioles, arterioles, and capillaries constitute the coronary microcirculation. Under baseline conditions, oxygen extraction from the arterial blood in the myocardium is close to 70%, and consequently, a rise in myocardial oxygen demand can only be met by a proportionate increase in coronary blood flow. Structural and functional alterations in the coronary microcirculation may contribute to and/or aggravate heart failure [34,35]. Beneficial effects of HDLs on the coronary microcirculation may therefore be a mediator on the causal pathway between HDL-targeted therapies and clinically relevant endpoints. HDLs exert potent effects on the endothelium. These effects occur via enhanced endothelial survival [36] and endothelial cell migration [37]. Interaction between HDLs and SR-BI initiates signaling in endothelium through the tyrosine-protein kinase Src, resulting in endothelial nitric oxide synthase activity and cell migration [37,38,39,40]. The PDZ domain-containing protein PDZK1 is expressed in the endothelium and is required for HDL activation of endothelial nitric oxide synthase and for cell migration [40]. SR-BI is a plasma membrane cholesterol sensor and proper function of the plasma cholesterol sensing domain is required for the activation of endothelial nitric oxide synthase and of endothelial cell migration in vitro [41].

HDLs also promote endothelial progenitor cell-mediated repair [42,43,44,45]. The effect of HDLs on endothelial cell migration is mediated via nitric oxide and increased nitric oxide production in these cells requires signaling via SR-BI and extracellular signal-regulated kinases [44]. Moreover, apo A-I enhances proliferation of endothelial progenitor cells and stimulates angiogenesis through the cell surface F1-ATP synthase, which is a high affinity receptor of apo A-I [46].

HDLs serve as cholesterol donors for caveolae via an SR-BI-dependent process [47]. Therefore, HDLs, by maintaining the lipid microenvironment, will inhibit subcellular redistribution and inactivation of endothelial nitric oxide synthase [47]. Enhanced nitric oxide production in endothelial cells induced by HDLs promotes endothelium-dependent vasodilatation [48]. Furthermore, HDLs enhance endothelial nitric oxide protein level in vascular endothelial cells by increasing its half-life [49]. These effects may counteract myocardial hypoxia in the setting of heart failure. Finally, HDLs may exert anti-inflammatory effects at the level of the myocardial endothelial cells. HDLs inhibit the expression of adhesion molecules induced by pro-inflammatory cytokines in endothelial cells [50]. These endothelial anti-inflammatory effects of HDLs are mediated by SR-BI, the SR-BI adapter protein PDZK1, and endothelial nitric oxide synthase [51]. Moreover, HDLs promote the expression of the anti-inflammatory cytokine transforming growth factor-β2 in endothelial cells [52]. 

The coronary microcirculation plays a key role in myocardial perfusion. Since endothelium–cardiomyocyte interactions affect cardiomyocyte function, the interaction of HDL particles with endothelial cells will not only have an impact on cardiac perfusion, but may also modulate the paracrine effects of the endothelium on cardiomyocytes. Regulation of cardiomyocyte function by nitric oxide, which may be potentiated by HDLs, takes place in both an autocrine and paracrine manner [53]. Modulation of the contractile state of subjacent cardiomyocytes by endothelial cells in the heart may occur both via the vascular endothelium in the myocardial capillaries and via the endocardial endothelium [54]. Consequently, HDLs may influence cardiac function not only via its impact on myocardial endothelial cells, but also via its effects on the endothelium of the endocardium.

Taken together, HDLs may result in beneficial structural effects on the myocardial vascular compartment, enhance endothelial function in the myocardium, and exert direct anti-inflammatory effects on the endothelium. HDLs may also modulate endothelium–cardiomyocyte interactions.

### 2.3. Direct Effects of HDLs on Fibrosis

Fibroblasts are the third most common cell type in the myocardium [16,17,18]. A properly organized extracellular matrix in a physiologically healthy heart contributes to synchronized contraction, tight cell–cell coupling, and directional action potential propagation [55]. Myocardial fibrosis results from the proliferation and activation of fibroblasts and myofibroblasts, leading to excess deposition of collagenous and non-collagenous extracellular matrix [56]. This process induces dysregulated organ architecture and function and is, besides capillary rarefaction, an important hallmark of pathological hypertrophy [57]. Myocardial fibrosis affects global tissue organization, leads to irregular action potential propagation, and reduced cardiac compliance, affecting diastolic function. This detrimental process may be counteracted by HDLs. First, HDLs reduced transforming growth factor-ß1-induced collagen accumulation in murine fibroblasts [58]. Moreover, HDLs decreased the amount of transforming growth factor-ß1 in the myocardium [59]. Finally, HDLs have been shown to diminish transforming growth factor-ß1-induced endothelial-mesenchymal transition in aortic endothelial cells in vitro [60]. In this respect, it should be pointed out that cardiac fibroblasts mainly originate from primary mesenchymal cells, but also from endothelial–mesenchymal transition [56,61]. On the other hand, fibroblasts are not only resident myocardial cells or derived from resident myocardial cells only, but can also originate from circulating fibrocytes [56]. Fibrocytes are circulating monocyte-derived cells that are precursors of fibroblasts and have the ability to differentiate into active fibroblasts [62]. The number of circulating fibrocytes increases in cardiovascular and chronic inflammatory disorders [63,64]. Furthermore, innate and adaptive immune cells promote the activation of fibroblasts and of myofibroblasts [56,65]. Therefore, the impact of HDLs on myocardial biology is not restricted to its effects on resident cells, but may also be mediated indirectly via systemic effects.

### 2.4. Anti-Inflammatory Effects of HDLs

Myocardial inflammation is considered to be a key player in the pathogenesis of heart failure since it is involved in adverse left ventricular remodeling [66,67], may drive myocardial fibrosis [56,65], and leads directly to cardiomyocyte dysfunction [68]. In particular, inflammation and immune cells play a key role in the pathogenesis of heart failure with preserved ejection fraction (HFpEF) [69,70,71]. Assembly of NLRP3 inflammasome complex in innate immune cells results in the cleavage and activation of inflammatory caspases and in the cleavage of pro-inflammatory cytokines into their mature bioactive species [72]. Activation of the NLRP3 inflammasome is terminated by HDLs [73]. Production of monocyte chemoattractant protein-1 production was also inhibited by HDLs [74]. Apo A-I impedes the activation of CD11b, an integrin family member, which pairs with CD18 to form the CR3 heterodimer and regulates leukocyte adhesion and migration [75]. Furthermore, neutrophil activation is impeded by HDLs and by apo A-I in vitro and in vivo [76]. HDLs also hinder the binding of T-cell microparticles to monocytes, which results in diminished production of pro-inflammatory cytokines [77]. Finally, HDLs inhibit interleukin-6 production and neutralize C-reactive protein proinflammatory activity [78,79].

HDLs may exert direct anti-inflammatory effects in the myocardium or systemic anti-inflammatory effects. The existence of a cardiosplenic axis has not only been demonstrated to exist in a model of chronic ischemic heart failure in mice [80], but splenic metabolic activity has also been shown to constitute a cardiovascular risk factor in humans [81]. Therefore, systemic immunomodulation by HDLs should be considered in the framework of clinical translation of HDL-targeted therapies [82].

Pathological conditions associated with systemic inflammation such as metabolic syndrome, chronic heart diseases, chronic kidney diseases, and several rheumatological disorders are marked by HDL dysfunction affecting the antioxidative and anti-inflammatory potential of HDLs [83,84]. HDLs may not only lose its normal function, but also acquire pathological properties such as proinflammatory effects [85,86]. This dysfunction is both the result of compositional changes of HDL particles and chemical modification of the protein and lipid content of HDLs [85].

### 2.5. Antioxidative Effects of HDLs

Oxidative stress emerges from an imbalance between the production of reactive oxygen species and inactivation of these species by antioxidative defense systems and represents a key player in the pathogenesis of heart failure [87,88]. Excessive release of reactive oxygen species may originate from xanthine oxidase, uncoupling of nitric oxide synthases, nicotinamide adenine dinucleotide phosphate (NADPH) oxidases, and electron leakage from the mitochondrial electron transport chain during oxidative phosphorylation [89,90,91]. Antioxidants and antioxidant defense systems may be significantly impaired in heart failure [92,93,94,95,96]. Reactive oxygen species can induce harm to DNA and RNA, cause protein damage, and affect the integrity of cell membranes. Aside from these direct detrimental effects, the indirect damage may result from the activation of proinflammatory and proapoptotic pathways by reactive oxygen species [97]. Overall, they may play a major role in ventricular remodeling by inducing cardiomyocyte dysfunction, cardiomyocyte cell death, and myocardial fibrosis [98]. 

The antioxidative potential of HDLs may significantly reduce systemic oxidative stress and oxidative stress in the myocardium via multiple mechanisms. Indirectly, reduced inflammation secondary to the anti-inflammatory potential of HDLs may reduce oxidative stress [85]. Oxidation products from LDLs may be transferred to HDLs so that HDLs serve as a ‘sink’ for oxidized lipids [99,100,101,102,103]. A significant part of the antioxidative effects of HDLs is mediated by apo A-I [102]. HDLs may also reduce oxidative stress by preventing endothelial nitric oxide uncoupling [104]. Furthermore, HDLs are a carrier of the antioxidative enzyme paraoxonase 1 [105,106,107]. Finally, platelet-activating factor acetylhydrolase is an HDL-associated enzyme that induces the hydrolysis of peroxidized fatty acids of phospholipids [108,109]. Since oxidized phospholipids are characterized by strong pro-inflammatory effects, platelet-activating factor acetylhydrolase activity may be a major contributor to the anti-inflammatory potential of HDLs. C57BL/6 human *apo A-I* transgenic mice are characterized by a drastic elevation of platelet-activating factor acetylhydrolase and paraoxonase activity compared to wild-type C57BL/6 mice [110]. 

## 3. High-Density Lipoproteins (HDLs) and Heart Failure: Epidemiological Perspective

Epidemiological models are only hypothesis-generating and causality cannot be inferred. In particular, low HDL cholesterol can be an integrated biomarker of adverse metabolic processes including abnormal metabolism of triglyceride-rich lipoproteins, insulin resistance, and ongoing tissue inflammation [10].

In participants of the Framingham Heart Study who were free of coronary heart disease at baseline, decreased HDL cholesterol levels were independently associated with the incidence of heart failure after adjustment for myocardial infarction and clinical covariables [111]. In addition, low HDL cholesterol levels and low plasma levels of apo A-I, the main apolipoprotein of HDL, have been associated with an unfavorable prognosis in patients with heart failure regardless of etiology [112,113]. Therefore, HDL cholesterol is both a predictor of heart failure incidence and of survival in patients with prevalent heart failure. 

**Figure 1:**
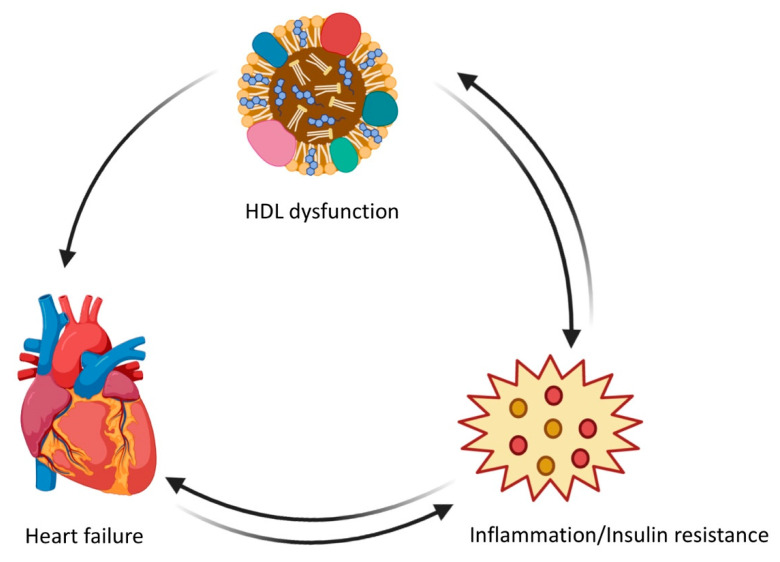
High-density lipoprotein (HDL) dysfunction in relation to inflammation/insulin resistance and heart failure: a pattern of cyclic causality.

As already mentioned, the cholesterol content of HDL particles does not necessarily mirror the biological function of HDLs. HDL dysfunction could be a contributing factor to both the onset and the progression of heart failure. First, inflammation and heart failure are strongly linked [114]. Secondly, heart failure has been associated with systemic insulin resistance. Both inflammation and insulin resistance can lead to HDL dysfunction [4]. HDL dysfunction and heart failure can thus mutually reinforce each other, in other words, there may be a pattern of cyclical causality (Figure 1). A case-control study demonstrated that the anti-oxidative and cholesterol efflux capacities of HDLs are reduced in patients with ischemic cardiomyopathy [115]. In a prospective cohort study of chronic heart failure patients, HDL antioxidative function was an independent predictor of the composite endpoint of death due to cardiovascular events and heart transplantation [116]. Moreover, no differences in the predictive value of HDL antioxidative function were detected between patients with ischemic and non-ischemic chronic heart failure [116]. Finally, levels of apo M, which mediates the physical interaction between HDLs and sphingosine-1-phosphate and exerts anti-inflammatory and cardioprotective effects in animal models, were inversely and independently associated in the Penn Heart Failure Study with the risk of death and the composite endpoint of death/ventricular assist device implantation/heart transplantation [117]. In particular, this association remained after adjustment for ischemic versus non-ischemic pathogenesis of heart failure.

## 4. HDLs and Heart Failure: Intervention Studies in Mouse Models

Scientific reductionism as applied in ex vivo or in vitro investigations reduces complex interactions and entities to the sum of their constituent parts and may lead to the discovery of biological mechanisms that are valid within the specific experimental context. However, knowledge of biological mechanisms is a weak and insufficient foundation for predicting a potential therapeutic effect. Rather, the integrated effect of HDL-targeted interventions on hard in vivo endpoints in animal models should be explored to provide stronger evidence for the potential of such therapies in terms of clinical translation. This point is, for example, illustrated by the fact that direct effects on contractility may be accompanied by indirect effects on myocardial fibrosis since mechanical cues affect myofibroblast differentiation [55,118,119]. Several recent studies in mouse models of non-ischemic heart failure have shown that HDLs may prevent heart failure development or induce reversal of existing heart failure, whereas dysfunctional HDLs may worsen heart failure development [59,120,121,122,123,124]. In all studies supporting this claim, there is a complete absence of coronary atherosclerosis, which implies that the effects of HDLs on cardiac structure and function and on heart failure are secondary to the direct effects of HDLs in the myocardium or secondary to its systemic anti-inflammatory and antioxidative properties or its effects on circulating cells. As already stated, the effect of HDL-targeted therapies on ventricular remodeling and cardiac dysfunction post-myocardial infarction [8,9,10] is outside the scope of the current review. 

Heart failure is marked by clinical symptoms and signs of increased tissue and organ water and of compromised tissue and organ perfusion. The diagnosis of heart failure in humans is and will remain a clinical diagnosis that is corroborated by functional, structural, and biomarker data. The diagnosis of heart failure in mice is not easy, since, like in humans, cardiac dysfunction itself is not the criterion for diagnosis. The operational criterion that can be used for diagnosis of heart failure in mouse models is an increased lung weight and/or a decreased exercise capacity in the presence of an objectified cardiac dysfunction.

### 4.1. Contrasting Effects of HDL Dysfunction and Apo A-I Gene Transfer on Heart Failure with Reduced Ejection Fraction

To illustrate the consistency of the experimental results, we will first discuss the effect of HDL dysfunction on cardiac function and heart failure. The HDL receptor SR-BI (scavenger receptor class B, type I) is an important regulator of lipoprotein metabolism and of whole-body cholesterol homeostasis [120,125]. SR-BI binds HDLs with high affinity and is predominantly expressed in the liver and non-placental steroidogenic tissues [126]. Selective cholesterol uptake by SR-BI in hepatocytes and subsequent excretion in the canaliculi is a major route of cholesterol secretion. *Scarb1^−/−^* mice, which lack SR-BI protein expression, are distinguished by an increased serum cholesterol and presence of enlarged HDL particles enriched in free cholesterol and apolipoprotein E [120,127]. Increased serum cholesterol in *Scarb1^−/−^* mice chiefly reflects a pronounced elevation of HDL cholesterol levels. However, the absence of hepatic SR-BI activity results in dysfunctional HDL particles marked by a reduced capacity to promote cholesterol efflux, a diminished antioxidative potential resulting in increased oxidative stress, and a reduced anti-inflammatory potential. Transverse aortic constriction is a commonly used technique to induce pressure overload in mice. Pressure overload results in cardiac hypertrophy, cardiac dilation, and heart failure with reduced ejection fraction (HFrEF) (ejection fraction <40%). In the presence of pressure overload due to transverse aortic constriction, cardiac hypertrophy is more pronounced in *Scarb1^−/−^* mice [120]. In addition, the degree of pathological ventricular remodeling in *Scarb1^−/−^* mice is increased with more pronounced interstitial fibrosis, more perivascular fibrosis, and a more prominent decrease in myocardial capillary density [120]. Finally, heart failure is more prominent in *Scarb1^−/−^* pressure overload mice as evidenced by the more pronounced increase in lung weight compared to wild-type mice. All these adverse effects of SR-BI deficiency were counteracted by SR-BI gene transfer, whereby this protein is stably and persistently expressed exclusively in the liver parenchymal cells (hepatocytes) and HDL metabolism is restored. In addition, HDL dysfunction in *Scarb1^−/−^* mice also leads to systolic and diastolic dysfunction in the absence of pressure overload. Cardiac function in *Scarb1^−/−^* mice without pressure overload is completely normalized when SR-BI expression is induced by gene therapy in the liver parenchymal cells [120].

These results substantiating the negative impact of HDL dysfunction should be contrasted with the impact of adeno-associated viral serotype 8 human apo AI (AAV8-AI) gene transfer in C57BL/6J low density lipoprotein receptor (LDLr)^−/−^ mice [121]. The lipoprotein profile of C57BL/6J LDLr^−/−^ on standard chow resembles the human lipoprotein profile in contrast to wild-type mice. AAV8-A-I gene transfer induces supraphysiological isovolumetric relaxation and thus supernormal diastolic function in mice without pressure overload. In the presence of pressure overload, not only is cardiac hypertrophy inhibited by AAV8-A-I gene therapy, but also pathological remodeling at the microscopic level as evidenced by the reduced interstitial and reduced perivascular fibrosis and the higher myocardial capillary density. An inhibitory effect of HDL infusion on cardiac hypertrophy induced by transverse aortic constriction has previously been demonstrated by Lin et al. [22]. In pressure overload mice, both systolic and diastolic cardiac function were improved by AAV8-A-I gene transfer and heart failure was prevented as indicated by the normal lung weight. Taken together, these findings on the effect of improved HDL function after AAV8-A-I gene transfer are the mirror image of the observations in *Scarb1^−/−^* mice characterized by HDL dysfunction.

### 4.2. Successful Treatment of Existing Heart Failure with Reduced Ejection Fraction in Mice by Treatment with Apo A-I_Milano_ Nanoparticles

The previous studies concern prevention studies in which gene transfer was performed two weeks before transverse aortic constriction and in which morphological, physiological, and clinical endpoints were analyzed eight weeks after the start of pressure overload. The more clinically relevant question is whether HDL-targeted interventions may be suitable for the treatment of existing heart failure.

Apo A-I_Milano_ is an apo A-I mutant that results from an arginine 173 to cysteine mutation [128,129]. This mutation was first described in a family from Limone sul Garda in Northern Italy in 1980 [128,129]. Heterozygous carriers of this apo A-I_Milano_ mutant in this family are characterized by higher life expectancy [130] and a much lower rate of atherosclerosis than expected based on their plasma levels of HDL cholesterol (in the lowest fifth percentile (10–30 mg/dL)) [131]. MDCO-216 is a form of reconstituted HDLs consisting of purified recombinant dimer apo A-I_Milano_ complexed with 1-palmitoyl-2-oleoyl-sn-glycero-3-phosphatidylcholine [132]. The clinical safety of MDCO-216 has been confirmed in multiple studies [132,133,134,135].

In mice with pre-existing heart failure induced by transverse aortic constriction, treatment with MDCO-216 (dose 100 mg /kg; five injections 48 h apart) induced normalization of lung weight, regression of interstitial fibrosis, increased relative myocardial vascularity, and improved isovolumetric relaxation. Both systolic and diastolic function were improved after treatment with apo A-I_Milano_ nanoparticles. Control buffer injection resulted in no effect [122].

### 4.3. Successful Treatment of Existing Heart Failure with Preserved Ejection Fraction in Mice by Treatment with Apo A-I_Milano_ Nanoparticles

Inhibition of the renin-angiotensin-aldosterone system and ß-receptor blockade improve survival and reduce hospitalization in patients with HFrEF [136]. In contrast to these advances in the treatment of HFrEF, no therapy has been found to be of clinical benefit in patients with HFpEF (ejection fraction ≥50%) and mortality in patients with HFpEF is unchanged [137,138]. This difference in outcome is not surprising since left ventricular dilation in HFpEF is a priori limited or absent [139]. HFpEF represents an important unmet therapeutic need. The efficacy of HDL-targeted treatments on a clinically relevant endpoint may therefore be more easily demonstrated in HFpEF patients.

However, developing adequate animal models of HFpEF is far from straightforward. HFpEF is not only caused by diastolic dysfunction, but is the manifestation of decreased ventricular diastolic reserve function, diminished ventricular systolic reserve function, decreased heart rate reserve, atrial dysfunction, pulmonary hypertension, and abnormalities in striated muscle [140]. Many of these anomalies are not present at rest, but become apparent during exercise [140]. Finally, the clinical HFpEF population is very heterogeneous.

We developed a HFpEF model by long-term (26 weeks) feeding of a diet containing 0.2% cholesterol and 10% coconut oil (CC diet) in female C57BL/6N mice [59]. Prior work had demonstrated that feeding this diet for eight weeks caused myocardial fibrosis and diastolic dysfunction in female C57BL/6N mice [141]. Long-term feeding of this diet for 26 weeks caused pathological left ventricular hypertrophy with a lower myocardial capillary density at the microscopic level and a markedly increased interstitial fibrosis compared to mice on standard diet. Systolic and diastolic function parameters determined with a pressure-volume catheter were significantly impaired in CC diet mice, resulting in decreased stroke volume, decreased cardiac output, and pathological ventriculo–arterial coupling. However, the ejection fraction was maintained. Eight intraperitoneal injections of MDCO-216 (100 mg/kg; interval 48 h) in CC diet mice resulted in decreased cardiac hypertrophy, increased capillary density, and decreased interstitial fibrosis [59]. Treatment with MDCO-216 resulted in fully normalized cardiac function while buffer injection had no effect. Exercise capacity was also significantly improved after treatment with MDCO-216 [59]. 

The efficacy of MDCO-216 treatment was also demonstrated in a mouse model of hypertension-associated HFpEF [124]. To induce hypertension and HFpEF, subcutaneous infusion of angiotensin II in combination with 1% NaCl in the drinking water was started at the age of 12 weeks in male C57BL/6N mice and was maintained for the entire duration of the experiment. MDCO-216 hypertension mice were treated with five intraperitoneal administrations of 100 mg/kg (protein concentration) of apo A-I_Milano_ nanoparticles (MDCO-216) at an interval of 48 h each. Treatment with MDCO-216 partially reversed established cardiac hypertrophy, cardiomyocyte hypertrophy, capillary rarefaction, and perivascular fibrosis in this model. Pressure-volume loop analysis was consistent with HFpEF in hypertension mice as evidenced by the preserved ejection fraction and a significant reduction of cardiac output. MDCO-216 completely reversed cardiac dysfunction and abolished heart failure as evidenced by the normalization of the lung weight and normal biomarkers of heart failure. In conclusion, apo A-I_Milano_ nanoparticles also constitute an effective treatment for established hypertension-associated HFpEF.

**Figure 2:**
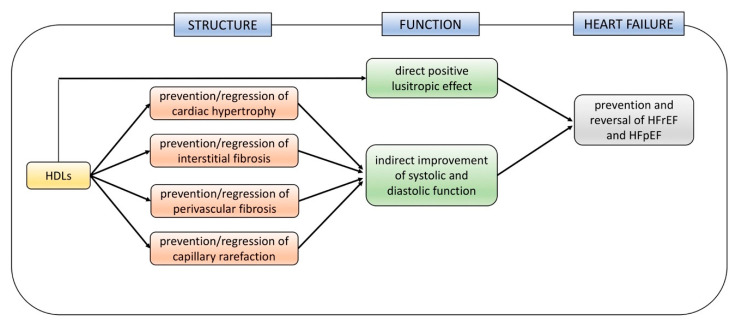
Effects of HDL-targeted therapies on cardiac structure, function, and heart failure in murine models of Heart Failure with Reduced Ejection Fraction (HFrEF) and Heart Failure with Preserved Ejection Fraction (HFpEF).

The global effects of HDLs on cardiac structure, function, and heart failure are summarized in Figure 2.

## 5. HDL-Targeted Therapies in Clinical Trials

Up until now, HDL-targeted therapies for heart failure have never been evaluated in patients with heart failure. Moreover, to date, it has never been demonstrated that HDL-targeted therapies result in a meaningful effect on a clinically relevant endpoint. Whereas the current review deals with HDL-targeted therapies and heart failure, it is useful to briefly discuss the current state-of-the-art of the HDL hypothesis. According to his hypothesis, increasing HDLs or improving HDL function will lead to a decrease in coronary events. This hypothesis has been investigated for nearly six decades. It should be kept in mind that there are hardly any HDL-specific interventions. The strict criterion for an HDL-specific intervention is that the causal path between therapeutic intervention and hard clinical endpoint is obligatory via HDLs [142]. This is not the case for several drugs that affect serum HDL cholesterol levels that were evaluated in phase III clinical trials with hard clinical endpoints (niacin, fibrates, cholesterol ester transfer protein inhibitors) [142]. The current ongoing AEGIS-II (Apo A-I Event Reduction in Ischemic Syndromes II) clinical trial is a formal test of the HDL hypothesis. In AEGIS-II, the effect of CSL112 (four intravenous infusions of 6 g CSL112 at one-week intervals) versus placebo (albumin solution) will be evaluated in patients with acute ST elevation myocardial infarction or non-ST elevation myocardial infarction. The primary endpoint is the composite of cardiovascular death, non-fatal myocardial infarction, and non-fatal stroke. CSL112 is a formulation of human apo A-I in which apo A-I is complexed with phosphatidylcholine to form disk-shaped HDL particles each consisting of two apo A-I molecules and about 110 molecules of phosphatidylcholine [143]. Infusion of CSL112 leads by fusion with circulating HDL_3_ particles and subsequent cleavage to several HDL species including preβ_1_-HDLs [144,145]. This study recruits 17,400 patients and results are expected in 2022. Specific inclusion criteria are aimed at enrichment with high-risk patients. Predicting the outcome of a randomized clinical trial is de facto difficult and this is certainly the case for AEGIS-II. The enthusiasm for the HDL hypothesis is mainly based on biological plausibility and on observational epidemiological data. Biological plausibility is a weak basis for predicting clinical reality and an inherent problem of observational epidemiological data is unmeasured and/or residual confounding (as a result of measurement errors). 

There are striking arguments against the HDL hypothesis [142]. Specifically, intervention studies using human *apo A-I* overexpression have failed to induce regression of advanced atherosclerosis in animal models, which contrasts with the pronounced regression effects of cholesterol lowering gene transfer [146,147,148,149]. The most striking argument against the HDL hypothesis are the results from two distinct Mendelian randomization studies comprising over 165,000 subjects that failed to demonstrate an association between genetically determined variation of HDL cholesterol and risk of myocardial infarction [150,151]. In contrast, Mendelian randomization studies support a causal effect of triglycerides, which inversely correlate with HDL cholesterol levels, on coronary heart disease risk [152]. In favor of a positive outcome of AEGIS-II, is the fact that the intervention in AEGIS-II is unique due to the specific population of cholesterol acceptors that are formed following the fusion and cleavage process. The great merit of this study is that the HDL hypothesis is effectively tested using a clinically relevant endpoint. After all, imaging biomarkers may be an unreliable surrogate of hard clinical endpoints [11]. Furthermore, Mendelian randomization studies do not constitute the equivalent of an intervention.

## 6. Conclusions

Intervention studies with HDL-targeted therapies in four different models of pre-existing heart failure (HFrEF after transverse aortic constriction [122], heart failure in a model of diabetic cardiomyopathy [123], two HFpEF models [59,124]) show that the impact of HDL-targeted interventions on cardiac structure and function is highly reproducible, indicating broad robustness of the effects. HDL-targeted interventions in these animal models result in a reversal of pathological cardiac hypertrophy with regression of myocardial fibrosis and of capillary rarefaction, in a powerful improvement of systolic and of diastolic cardiac function, and in a reversal of heart failure. The observed effects are consistent with in vitro and ex vivo data evaluating the effect of HDLs on cardiomyocytes, endothelial cells, fibroblasts, and myofibroblasts, and are concordant with the antioxidative potential of HDLs. Until now, HDL-targeted therapies for heart failure have never been evaluated in patients with heart failure. On the other hand, the safety of administration of apo A-I nanoparticles has already been extensively documented in humans. In contrast to advances in the treatment of HFrEF, no therapy has been found to be of clinical benefit in patients with HFpEF. As HFpEF represents an important unmet therapeutic need, it is likely to be the preferred therapeutic domain for the further development of HDL-targeted interventions.
